# The influence of familiarisation and item repetition on the name agreement effect in picture naming

**DOI:** 10.1177/17470218241274661

**Published:** 2024-09-12

**Authors:** Ruth E Corps, Antje S Meyer

**Affiliations:** 1 Department of Psychology, University of Sheffield, Sheffield, UK; 2 Psychology of Language Department, Max Planck Institute for Psycholinguistics, Nijmegen, The Netherlands; 3 Radboud University, Nijmegen, The Netherlands

**Keywords:** Speech production, lexical access, picture naming, name agreement, familiarisation, repetition priming

## Abstract

Name agreement (NA) refers to the degree to which speakers agree on a picture’s name. A robust finding is that speakers are faster to name pictures with high agreement (HA) than those with low agreement (LA). This NA effect is thought to occur because LA pictures strongly activate several names, so speakers need time to select one. HA pictures, in contrast, strongly activate a single name, so there is no need to select one name out of several alternatives. Recent models of lexical access suggest that the structure of the mental lexicon changes with experience. Thus, speakers should consider a range of names when naming LA pictures, but the extent to which they consider each of these names should change with experience. We tested these hypotheses in two picture-naming experiments. In Experiment 1, participants were faster to name LA than HA pictures when they named each picture once. Importantly, they were faster to produce modal names (provided by most participants) than alternative names for LA pictures, consistent with the view that speakers activate multiple names for LA pictures. In Experiment 2, participants were familiarised with the modal name before the experiment and named each picture three times. Although there was still an NA effect when participants named the pictures the first time, it was reduced in comparison to Experiment 1 and was further reduced with each picture repetition. Thus, familiarisation and repetition reduced the NA effect but did not eliminate it, suggesting speakers activate a range of plausible names.

## Introduction

A robust finding in picture-naming studies is the name agreement (NA) effect: Speakers are faster to name pictures that are named in the same way by most other speakers in the same sample (e.g., most people refer to a picture of a dog as *dog*; high NA) than pictures that elicit several different names, such as synonyms (e.g., *sofa* and *couch*) or hypernyms (e.g., *bike* and *racing bike*; low NA; e.g., Barry et al., 1997). A common assumption in the literature is that population-level norms (i.e., how others might name a picture) are represented in an individual speaker’s mind (see Balatsou et al., 2022, for discussion). A low agreement (LA) picture strongly activates several names, so speakers need time to select one. A high agreement (HA) picture, in contrast, strongly activates a single name, so there is no need to select one name out of several alternatives. As a result, HA pictures are named faster than LA pictures.

Recent models of lexical access suggest that the structure of the mental lexicon is flexible and changes with experience, such as repeated encounters with a word (e.g., Damian & Als, 2005; Howard et al., 2006; Oppenheim et al., 2010), and so the extent to which an object evokes different names and how strongly each of them is activated should change with experience. For example, Oppenheim et al. proposed that lexical accessibility changes across naming episodes as a result of the strengthening and weakening of connections between conceptual input and lexical nodes. In particular, the connection between the conceptual input and the selected lexical node is strengthened after naming so that it becomes more accessible for future retrieval, whereas the link between the conceptual input and coactivated (but non-selected) words is weakened so that these words are less accessible for future retrieval.

Thus, speakers should consider a range of names when naming pictures with LA, and the extent to which they consider each of these names should be altered by experience. These hypotheses—that LA pictures evoke several names in an individual speaker, and that they do so to different extents depending on experience—are plausible, but they have rarely been directly tested. We addressed them in two picture-naming experiments, in which participants named LA and HA pictures. In Experiment 1, participants named each picture once and we tested, first, whether LA pictures were named faster than HA pictures (including both modal and alternative names), and, second, whether participants were faster to produce modal names (produced by the majority of the participants in the sample) than alternative but plausible names (produced by the minority) for LA pictures. The second result should occur if modal names are activated and compete for selection with alternative names. In Experiment 2, participants were familiarised with the modal name before the experiment and then named each picture three times. Thus, we tested whether current word activation levels were affected by previous activity in the lexical network (e.g., Oppenheim et al., 2010). As a further test of this hypothesis, we also conducted a cross-experiment analysis, comparing naming latencies with and without familiarisation.

In the sections that follow, we review the evidence suggesting that population-level NA is represented in an individual speaker’s mind. We then consider how repetition and familiarisation could affect the NA effect, with the goal of understanding how the NA effect is affected by exposure. Finally, we describe our study and our predictions in more detail.

### Population-level NA

NA is a population-level variable: It is defined as the degree to which participants in a sample agree on how to name a picture. A common assumption in accounts of the NA effect is that population-level norms (i.e., how others might name a picture) are represented in an individual’s speaker’s mind. For example, if different speakers spontaneously use *bike*, *bicycle*, and *racing bike* to name a picture of a vehicle that has two wheels and is moved by foot pedals, then it is assumed that each individual speaker considers this range of responses and selects one when they name the picture. As a result, speakers are slower to name LA pictures than HA pictures, which have only one plausible name.

The assumption that population-level norms are represented in an individual speaker’s mind has rarely been directly tested. In a recent study, Balatsou et al. (2022) examined how likely participants were to use modal and alternative names when they named HA and LA pictures twice. As expected, participants were more likely to produce modal than alternative names on both presentations of the picture. In addition, participants tended to use the same name twice: their name choice on the picture’s second presentation was predicted by their name choice on the first presentation. However, when participants did switch names, the likelihood of switching depended on the NA of the names used in the two sessions. In particular, participants were more likely to switch from an alternative to a modal name for pictures with HA compared to those with LA. Furthermore, when participants switched from a modal to an alternative name, they were more likely to switch to a strong alternative name than to a weak alternative name. This pattern suggests that speakers have individual preferences for certain names (idiolects), but they also represent the strength of population-level NA, which predicts the amount of conflict between the names and makes LA names harder to access.

Balatsou et al. only examined which object names participants chose. We extend this work in Experiment 1 and compare naming latencies for modal and alternative names. In psycholinguistic studies, naming latencies are typically only analysed for correct (or modal) responses, given that incorrect and alternative responses tend to represent a small subset of the data. However, many alternative responses are expected when a study involves LA pictures, particularly if participants are not familiarised with the materials and their names. In some NA studies (e.g., Britt et al., 2016; Park & Gabrieli, 1995), all valid picture names were included in the analysis, but the latencies for modal and alternative names were not compared. This analysis is important because it tells us whether the NA effect occurs because individual speakers represent population-level norms. If, contrary to Balatsou et al.’s argument, NA simply reflects a sampling of each speaker’s individual preferences with some speakers only considering one name of a picture (e.g., *bike*) and others only considering another name (e.g., *racing bike*), then (everything else being equal) we expect no difference in naming latencies for modal and alternative names. Alternatively, if population-level NA is represented in the individual speakers’ minds and biases them towards the choice of modal names, then modal names should be produced faster than alternative names.

### NA and experience

According to flexible models of lexical access, which allow for changes in lexical activation patterns over time and experience, the extent to which speakers activate alternative picture names should be affected by previous experience of producing them (e.g., Damian & Als, 2005; Howard et al., 2006; Oppenheim et al., 2010). These models predict that a word activated and selected previously will be more accessible for future retrieval than unselected but activated words. Thus, the NA effect should decrease with experience because alternative names for LA pictures should become less accessible.

In our study, we considered two forms of previous experience: repeated naming of the same picture and familiarisation with the picture and its name. We focused on these two sources of experience because many NA experiments (and picture-naming experiments in general) have participants name pictures multiple times and/or begin with a familiarisation phase, in which participants see the pictures they will name and read or hear the picture names they should produce. Thus, these manipulations allow us to test whether experience affects the NA effect, making a theoretical contribution to flexible models of lexical access, and a methodological contribution to the word production literature. If repetition and familiarisation reduce the NA effect, then researchers may wish to use these procedures when NA is a control variable, but not if it is the main variable of interest. In the following, we first consider the effect of repetition on the NA effect before considering familiarisation.

### NA and repetition

Research has shown that picture-naming latencies decrease when participants name the same pictures repeatedly (repetition priming; see, e.g., Francis, 2014, for a review). Moreover, this repetition priming effect can interact with other factors that affect naming latencies, such as the frequency and age of acquisition of the picture names, with pictures that are harder to name typically benefitting from repetition more than easier ones (e.g., Corps & Meyer, 2023; Griffin & Bock, 1998; Wheeldon & Monsell, 1992). The interaction between repetition and NA effects on picture-naming latencies has not been systematically studied. Park and Gabrieli (1995) found a stronger NA effect the first time participants named a set of pictures compared to when they named them again, but NA was not the main concern of this study and little information was provided about the pictures. In another study, Alario et al. (2004) asked participants to name the same pictures twice and found NA effects in both sessions of the experiment. However, they did not compare the size of the NA effect across sessions.

Furthermore, some studies suggest that competition between alternative names may not be reduced by repetition priming, contrary to the predictions of flexible models of lexical access. Kurtz et al. (2018; see also Jescheniak et al., 2017) tested whether words selected for production were more accessible for future retrieval than unselected but activated words. They used a picture-word interference (PWI) paradigm, in which participants named target pictures using their subordinate-level name (e.g., *poodle*) while ignoring distractor words that were phonologically related to the picture’s basic-level alternative name (e.g., *doll* for dog; Experiments 1 and 3), or they named pictures using their basic-level name (e.g., *dog*) while ignoring distractors that were phonologically related to the subordinate-level alternative name (e.g., *pool* for poodle; Experiment 2). Participants were slower to name pictures that were accompanied by a distractor word that was phonologically related to the target picture’s alternative name than unrelated distractors. Importantly, this mediated semantic interference effect was stable across many presentations of the same pictures, suggesting that the indirectly activated alternative remained an active competitor for naming.

In another study, Wöhner et al. (2024) found similar results. But they also investigated whether the same pattern occurred when target pictures (e.g., *duck*) were accompanied by distractor words from the same semantic category (e.g., *eagle*) or unrelated distractor words, thus studying a direct semantic interference effect (Experiment 2). This target–distractor relationship led to an interference effect, which was reduced with repeated naming. As the authors discuss, the reason for the discrepancy between direct and indirect semantic interference effects is unclear. However, the results do suggest that the semantic category coordinate became less accessible with repetition of the items. Similar results have been observed in other semantic interference studies (e.g., Bürki et al., 2020; Miozzo & Caramazza, 2003, Experiment 6).

Thus, there is some evidence that activated but non-selected words may become less accessible with repeated naming. We further investigated this issue in Experiment 2 by assessing whether competition from alternative names for LA pictures would be reduced by repetition. To do so, we had participants name each picture three times during the experiment, thus examining competition from activated but not selected names without having to use distractor words.

### NA and familiarisation

In addition, participants in Experiment 2 were familiarised with the pictures and their names before naming them. Familiarisation is often used in picture-naming studies to minimise the occurrence of missing responses, which can occur because the participant does not recognise the picture and responds incorrectly (e.g., naming a picture of an apple as *orange*) or does not respond at all. Familiarisation also encourages participants to respond uniformly (e.g., Bock, 1996), facilitating automatic measurement of naming latencies. But although familiarisation has these obvious practical benefits, it may also affect the NA effect because participants are told how they should name each picture. As a result, familiarisation may strongly prime one of the object names in the LA condition and reduce competition from alternative names, much like repeated naming. Alternatively, although less likely, familiarisation could increase the NA effect because participants are sometimes required to produce names they would not spontaneously use. For instance, if a picture evokes several names and the most common one is only chosen by 20% of participants then most speakers will be asked to use a dispreferred name. Producing this dispreferred name might hinder speakers compared with a situation where they can use their preferred name.

Although familiarisation may interact with the NA effect, research suggests it does not eliminate it completely. Previous studies have observed the NA effect both with familiarisation (e.g., Shao et al., 2014; Valente et al., 2014) and without it (e.g., Cheng et al., 2010; Vitkovitch & Tyrrell, 1995). However, these studies have not directly assessed the effect of familiarisation on the size of the NA effect. Doing so is important, both for providing insight into whether previous experience reduces competition from alternative names and for the design of naming studies. If familiarisation strongly reduces the NA effect, it suggests that previous experience with a picture and its modal name can affect the activation of alternative names during later naming. It also suggests that researchers may include a familiarisation phase in their study to eliminate unwanted confounding effects of NA. By contrast, if NA is an independent variable of interest, researchers might opt against familiarisation to avoid weakening the impact of this variable, even if doing so leads to more data loss. To investigate how familiarisation affects the NA effect, we compared naming latencies in Experiment 1 (without familiarisation) to naming latencies for the first presentation of the pictures in Experiment 2 (with familiarisation).

### The current study

In sum, we conducted two picture-naming experiments to investigate (1) whether individual speakers represent population-level NA and consider a range of names by comparing naming latencies for modal and alternative names and (2) whether the extent to which speakers consider different names is affected by previous experience by testing how the NA effect is affected by repetition and familiarisation. In both experiments, participants named LA and HA pictures. In Experiment 1, they named each picture once and we tested whether participants were faster to produce modal (produced by the majority of the participants in the sample) than alternative but plausible names (produced by the minority) for LA pictures, which should be the case if population-level NA biases them towards the modal name. In Experiment 2, participants named each picture three times and they were familiarised with the modal name before the experiment. Thus, we tested whether current word activation levels were affected by previous activity in the lexical network (e.g., Oppenheim et al., 2010). As a further test of this hypothesis, we also conducted a cross-experiment analysis, comparing naming latencies with and without familiarisation.

## Experiment 1

### Participants

Prior to data collection, we conducted a power analysis using simr (version 1.0.6; Green & MacLeod, 2016) to determine our sample size. We used the condition means (HA = 882 ms; LA = 1074 ms) and standard deviations (HA = 90 ms; LA = 151 ms) from Vitkovitch and Tyrrell’s (1995) study as they did not familiarise participants with the picture names. We simulated a random data set of 60 participants with 75 HA and 75 LA pictures and then determined the power of observing an effect across 1,000 simulations. With 60 participants we reached a power estimate of 100% (95% confidence interval: 99.63, 100.00) for detecting an NA effect of 192 ms.

As a result, we recruited 61 native Dutch speakers from the Max Planck Institute participant database (44 females, 17 males; *M*age = 23.95 years), who participated in exchange for six euros. All participants lived in The Netherlands and had no known speaking, reading, or hearing impairments. Ethical approval for this study was given by the Ethics Board of the Social Sciences Faculty at Radboud University. We discarded data from 10 participants due to technical errors (mainly with recording the beep that marked trial onset; see Procedure), and so the analysis concerned data from 51 participants (35 females, 16 males; *M*age = 26.47 years). Note that with 51 participants, we still had 100% power to detect an NA effect of 192 ms (95% confidence interval = [99.63, 100.00]).

### Materials

We selected 150 colour pictures from the Dutch Bank of Standardised Stimuli (BOSS: Decuyper et al., 2021), which is a database of coloured photographs of everyday objects (see online Supplementary Material, Table A1 for a full list of items). The pictures were from 22 different categories (including animals, food, clothing, and furniture) determined by a native Dutch speaker (see the online Supplementary Material, Table A2). Half of the pictures had HA, whereas the other half had LA. We calculated modal NA, which was the percentage of participants who provided the most common name for a particular picture. Modal NA is higher when more participants provide the same name for a picture. We also calculated each picture’s H-value, which is defined as:

$$H=\sum_{k}^{i=1}P_{i}\log_{2}\left(\frac{1}{P_{i}}\right)$$

here, *k* is the number of different names that were given to a picture, and *P_i_* is the proportion of participants that used each of these names. The *H*-value is thus sensitive to the number of different names participants gave to a picture and the spread of responses (i.e., whether all names are equally frequent or one dominates; e.g., Snodgrass & Vanderwart, 1980). The closer the *H*-value is to zero, the more participants tend to agree on the picture’s name and the higher its NA. The LA and HA conditions differed significantly in modal NA, *t*(148) = 67.82, *p* < .001, and *H*-values, *t*(148) = −31.66, *p* < .001; see Table 1.

**Table 1.** Maximum, minimum, mean, and standard deviation (*SD*) of descriptive statistics for high and low agreement pictures from the BOSS database.

	High agreement				Low agreement			
	Max	Min	Mean	*SD*	Max	Min	Mean	*SD*
Do not recognise object (%)	7	0	0.55	1.20	3	0	0.30	0.70
Do not know the object’s name	10	0	0.80	1.95	5	0	1.15	1.44
*H*-value	0.30	0.00	0.11	0.11	2.88	0.98	1.86	0.47
Modal NA (%)	100	96	98	2	58	27	43	7
SUBTLEX frequency (occurrences per one million words)	311.67	0.11	16.01	43.18	247.48	0.05	12.83	34.30
Age of acquisition (years)	11.89	3.73	6.50	1.61	11.78	3.95	6.98	1.87
Word prevalence (*z*-score)	1.96	1.62	1.85	0.09	1.96	1.37	1.83	0.13
Word prevalence (%)	100	97	99	0.01	100	94	99	0.01
Object agreement (5-point rating)	4.88	3.72	4.32	0.27	4.85	3.68	4.25	0.27

We matched the pictures in the two conditions for SUBTLEX word frequency of the dominant names, *t*(134) = 0.47, *p* = .64, which is the frequency per million words from the SUBTLEX-NL database (Keuleers et al., 2010), and for age of acquisition, *t*(116) = −1.48, *p* = .14, which is the age the word was acquired (taken from Brysbaert et al., 2014). The two conditions did not differ in word prevalence, *z*-scores; *t*(124) = 1.15, *p* = .25, which is the percentage of a sample who knows a word, taken from a large online study by Keuleers et al. (2015). The two conditions also did not differ in the percentage of participants who did not recognise the object, *t*(148) = 1.57, *p* = .12, or did not know the object’s name, *t*(148) = −1.25, *p* = .21, when tested in Decuyper et al. (2021). As the table shows, both of these values were very low for the HA and LA objects. Finally, the two conditions did not differ in object agreement, measured by ratings on a 5-point scale for how well each image represented the actual concept, *t*(148) = 1.66, *p* > .10.

### Procedure

The experiment was administered online and remotely (i.e., in the participants’ own home) using Frinex (FRamework for INteractive EXperiments, a software package developed for running online experiments by the technical group at the Max Planck Institute for Psycholinguistics). Participants were encouraged to complete the experiment in a quiet environment, away from any distractions such as phones or television. Each trial began with a black fixation cross (+) presented in the centre of the screen for 500 ms, which coincided with the auditory presentation of a beep that was also presented for 500 ms. The timing of item presentation could differ for participants depending on their internet connection, and so this beep was used to mark the beginning of the trial and would later be used for measuring naming latencies. The fixation cross then disappeared, and the target picture was presented in the centre of the screen, at a size of 10 viewpoint height by 10 viewpoint width. After naming the picture, participants clicked on a “Volgende” (“next”) prompt at the bottom of the screen to begin the next trial. The next trial began 1,000 ms later. Participants were instructed to name the picture as soon as it appeared on-screen, and were explicitly told that there were no right or wrong answers.

Before beginning the main experiment, participants checked that their microphone was recording by naming a picture of a pizza. They then listened to the audio playback to ensure that they could clearly hear themselves and the beep. Participants completed four practice trials to familiarise themselves with the experimental procedure and then began the main experiment. They were given the opportunity to take a break halfway through (i.e., after 75 trials). The presentation order of the pictures was randomised for each participant.

### Results and discussion

#### Correspondence between norms

We used pictures from the BOSS database, which were normed for NA in a typed picture-naming task with approximately 50 participants per picture. Before comparing naming latencies for LA and HA pictures, we examined how well the norms from the BOSS data set corresponded to NA in our study, given that our participants responded verbally rather than with a typed response. We computed the *H*-values for each picture based on our participants’ responses and correlated them with the *H*-values in the BOSS database. We found a strong significant correlation between *H*-values (*R* = .91, *p* < .001) and the average *H*-values were similar in the two data sets for both the HA (BOSS *M* = 0.11, *SD* = 0.11; Data *M* = 0.34, *SD* = 0.40) and LA items (BOSS *M* = 1.86, *SD* = 0.47; Data *M* = 1.91, *SD* = 0.60). To determine whether items that were categorised as LA or HA based on the BOSS norms fell into the same category based on our data, we calculated the minimum *H*-value for the LA items using the BOSS norms (0.98) and then determined the number of HA items that had a higher *H*-value in the data norms (i.e., HA items that should be LA) and the number of LA items that had a lower *H*-value (i.e., LA items that should be HA). Using this approach, we recategorised seven of the original HA items as LA items and five of the original LA items as HA items for the following analyses giving us 73 HA items and 77 LA items. The items that were recategorised are marked as such in the online Supplementary Material.

Finally, 97.26% of the HA items had the same modal name in both data sets, whereas only 63.63% of the LA items had the same modal name. Thus, the classification of items as HA or LA was largely confirmed by our data, but modal names were often different for the LA items. As a result, we defined modal and alternative names using our data rather than the BOSS names when we compared naming latencies for modal and alternative names.

#### Testing for an NA effect

In our next analysis, we determined whether naming latencies differed for HA and LA items. Naming latencies were measured from picture onset (which co-occurred with beep onset) and were calculated as the difference between beep onset and speech onset +500 ms for beep duration. Audio recordings were annotated in Praat (Boersma & Weenink, 2001) by trained native Dutch speakers. We discarded 318 trials (4.16%) because we could not determine what the participant said or because they produced a non-speech sound, a disfluency, or an utterance repair. We also discarded 57 (0.75%) naming latencies longer than 4,000 ms because these were clear outliers. Participants produced an incorrect name, which did not match the picture and was not a plausible alternative name (e.g., producing *orange* to refer to a picture of an apple), on 79 of the HA trials (1.03%) and 221 of the LA trials (2.88%). As there were so few incorrect responses, we excluded these from further analysis. This left us with 6,975 trials for analysis. Raw data and analysis scripts are available at: https://osf.io/5kzuv/.

We evaluated the effects of NA on naming latencies (including both modal and alternative names) using linear mixed-effects models (Baayen et al., 2008) using the *lmer* function of the *lme4* package (version 1.1-26; Bates et al., 2021) in RStudio (version 1.2.5042). Naming latencies were predicted by NA (reference level: low vs. high), which was contrast-coded (−0.5, 0.5). We used the maximal random effects structure justified by our data (Barr et al., 2014), including by-participant random effects for NA and by-item intercepts. Correlations among random effects were set to zero to aid model convergence.

**Figure 1.**
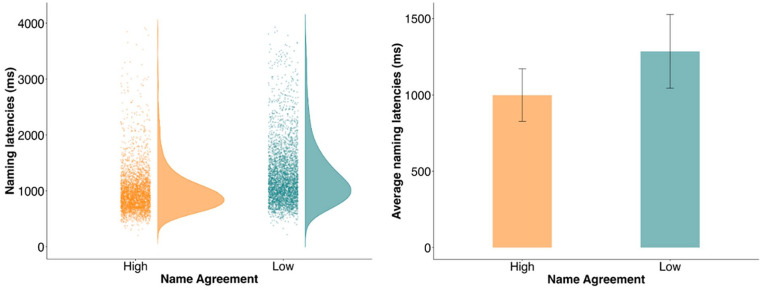
The left panel shows the distribution of naming latencies for high and low agreement pictures in Experiment 1, with dots showing individual data points for each NA condition. The right panel shows the mean naming latencies for the high and low agreement pictures, with error bars representing +1 standard deviation from the mean.

On average, participants responded 1,139 ms (*SD* = 499 ms) after picture onset. Participants were faster to name pictures with HA (*M* = 1,000 ms, *SD* = 173 ms) than those with LA (*M* = 1,286 ms, *SD* = 240 ms; *b* = −286.04, *SE* = 33.72, *t* = −8.48; see Figure 1). Thus, there was an NA effect even when participants were not familiarised with the pictures and could produce whichever name they wished, consistent with previous studies (e.g., Alario et al., 2004; Vitkovitch & Tyrrell, 1995).

#### Comparing latencies for modal and alternative names

**Figure 2.**
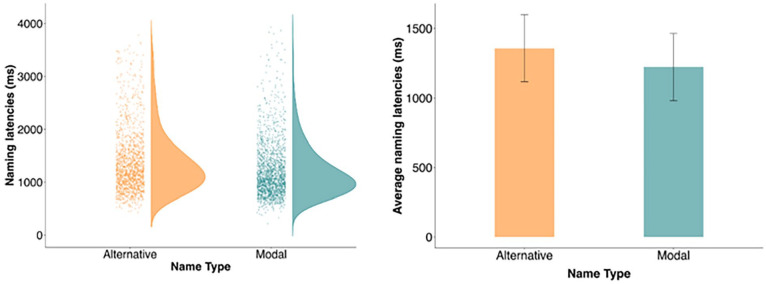
The left panel shows the distribution of naming latencies for alternative and modal names for low agreement pictures, with dots showing individual data points. The right panel shows the mean naming latencies for the alternative and modal names, with error bars representing +1 standard deviation from the mean.

Next, we examined whether naming latencies were faster for modal than alternative names. For this analysis, we focused on the LA pictures only because participants almost always produced the modal name for HA pictures (*M* = 96.96%; *SD* = 17.16%), but not for the LA pictures (*M* = 53.69%, *SD* = 49.87%). Naming latencies were predicted by Name Type (reference level: alternative vs. modal), which was contrast-coded (−0.5, 0.5), and we included by-participant and by-item random intercepts. We did not include random slopes because doing so resulted in a singular fit error. Correlations among random effects were again set to zero to aid model convergence. Participants were faster to produce modal names (*M* = 1,223 ms, *SD* = 242 ms) than alternative names (*M* = 1,357 ms, *SD* = 241 ms; *b* = −115.81, *SE* = 16.19, *t* = −7.15; see Figure 2).

To determine whether this difference occurred because alternative names were harder to access than modal names, we determined the word frequency, word prevalence, and age of acquisition (which are variables typically related to a word’s accessibility; see, e.g., Jescheniak & Levelt, 1994) of each alternative name that participants produced using the same norms as for the modal names (see Table 2 for descriptives). Modal and alternative names did not differ in age of acquisition, *t*(98) = 0.33, *p* = .75, but modal names had higher word prevalence, *t*(219) = −2.12, *p* = .04, and word frequency, *t*(293) = −2.01, *p* = .05, than the alternative names. As a result, we fitted a model comparing naming latencies for modal and alternative names (as in our previous analysis) but included centred word frequency and prevalence as covariates (i.e., fixed effects). We included by-participant and by-item random intercepts but did not include any random slopes because doing so resulted in a singular fit error.

**Table 2.** Maximum, minimum, mean, and standard deviation of descriptive statistics for modal and alternative names for low agreement pictures in Experiment 1.

	Modal names				Alternative names			
	Max	Min	Mean	*SD*	Max	Min	Mean	*SD*
SUBTLEX frequency (occurrences per one million words)	2612.54	0.02	59.90	313.69	818.90	0.02	14.03	78.21
Age of acquisition (years)	11.37	4.01	7.86	1.83	11.95	3.18	7.96	1.69
Word prevalence (*z*-score)	1.96	1.37	1.85	0.11	1.96	0.10	1.80	0.22

The full list of alternative names and their frequency of occurrence can be found at https://osf.io/5kzuv/. Participants were faster to produce names with higher word prevalence (*b* = −247.00, *SE* = 96.56, *t* = −2.56) but naming latencies were not affected by word frequency (*b* = −0.01, *SE* = 10.19, *t* = −0.00). Importantly, and as in our previous analysis, participants were faster to produce modal than alternative names (*b* = −98.10, *SE* = 20.27, *t* = −4.84), suggesting that this difference cannot be attributed entirely to differences in word frequency or word prevalence. One account of this finding is that each speaker considered the range of possible responses within the wider population and experienced difficulty when they produced a name that was dispreferred by this population, consistent with Balatsou et al.’s (2022) proposal. We return to this finding in section “General discussion” and consider alternative explanations.

## Experiment 2

Experiment 1 demonstrated that participants were faster to name HA than LA pictures, even when they were not familiarised with the pictures or their names. In Experiment 2, participants were familiarised with the pictures and their names and then named each picture three times. The latencies for the first presentation of the pictures show the NA effect after familiarisation and can be directly compared with the latencies observed in Experiment 1. Comparisons of the latencies across the three presentations provide information about the impact of repetition on the NA effect.

### Method

#### Participants

A further 59 participants were recruited using the same criteria as in Experiment 1 (46 females, 10 males, 3 NA; *M*age = 25.88 years) and participated in exchange for 10 euros. These participants did not complete Experiment 1. We discarded data from five participants due to technical errors (primarily because their audio recordings were empty), and so the analysis focuses on data from 54 participants (41 females, 10 males, 3 NAs; *Mage* = 25.85 years). Participants were recruited from the same pool as those in Experiment 1 and were similar in age and gender distribution.

#### Materials and procedure

We used the same materials and procedure as Experiment 1, but before naming the pictures participants were familiarised with them and their names. In this familiarisation phase, each trial began with a fixation cross (+) presented on the screen for 500 ms. After a blank interval of 300 ms, the picture was displayed in the centre of the screen, with the name participants should use in the main experiment presented beneath it. Participants were familiarised with the picture’s modal name from the BOSS norms. The picture and the name stayed on-screen for 3,000 ms and the next trial began automatically.

After this exposure phase, we tested participants’ knowledge of the picture names. Each trial began with a fixation cross (+) presented for 500 ms. The picture was displayed in the centre of the screen 300 ms later. Participants were instructed to type the name of the picture they had been familiar with into a text box presented below the picture. The picture remained on-screen until participants pressed a “Submit” button beneath the textbox. The picture then disappeared and the correct name was presented 300 ms later (in the format: “The correct name is: [picture name used in familiarisation]”). The name remained on-screen for 3,000 ms, and the next trial automatically began 1,500 ms later. In both of these phases, pictures were randomised using the same procedure as Experiment 1. Participants provided the correct (familiarised, modal) name 88% of the time, suggesting training was successful.

Participants then began the main experiment. The instructions were identical to Experiment 1, with the exception that participants were instructed to name the pictures using the names they learned during familiarisation. Each picture was presented three times. The pictures were randomised such that presentations of the same picture were separated by at least 20 trials (Jescheniak & Levelt, 1994). Before beginning the naming phase, participants checked their microphone was working, as in Experiment 1.

### Results and discussion

#### Testing for an NA effect

Naming latencies were measured from picture onset^1^ and were manually annotated using the same procedure as in Experiment 1. Before analysis, we discarded data from one picture (table tennis table) because participants were familiarised with a non-word in Dutch (*tafeltennistable* rather than *tafeltennistafel*), which may have confused them (162 trials; 0.67%). We discarded 252 trials (1.04%) because we could not determine what the participant said or because they produced a non-speech sound, a disfluency, or an utterance repair, and 816 trials (3.35%) because the recording of the picture name was cut-off and incomplete. We also discarded 46 (0.19%) naming latencies longer than 4,000 ms and five (0.02%) naming latencies less than 200 ms. Participants produced an incorrect name in 33 of the HA trials (0.14%) and 52 of the LA trials (0.21%). As there were so few incorrect responses, we excluded these from further analyses.

Participants almost always produced the modal name for the HA pictures (*M* = 99.74%, *SD* = 0.05%) and often produced it for the LA pictures (*M* = 95.15%, *SD* = 0.21%). Thus, we did not conduct an analysis comparing latencies for modal and alternative names in this experiment because the alternative names represented such a small subset of the data. Instead, we excluded these alternative responses from our analysis (30 cases for HA pictures and 549 cases for LA pictures). This left us with 22,355 trials for analysis.

To determine whether participants were faster to name HA than LA pictures, we analysed our data using the same procedure as for Experiment 1. But we also included continuous (numeric) presentation and its interaction with NA to determine whether naming latencies decreased with repeated naming and whether the NA effect was reduced by repeated naming. We initially fitted a model using the maximal random effects structure but including by-participant random effects for the interaction between NA and presentation resulted in convergence issues. As a result, we included by-participant random effects for NA and presentation and by-item random effects for presentation. Correlations among random effects were again set to zero to aid model convergence.

**Figure 3.**
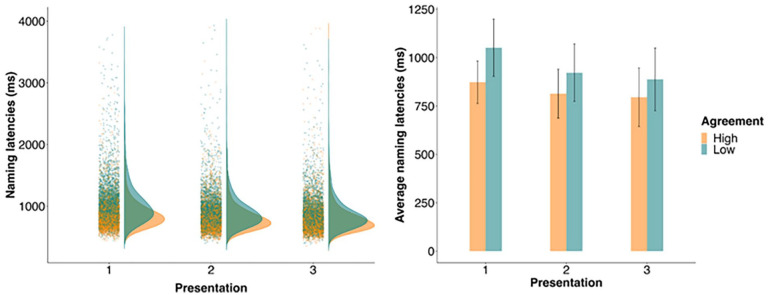
The left panel shows the distribution of naming latencies for HA and LA pictures on each of the three presentations in Experiment 2, with dots showing individual data points for each Name Agreement condition on each presentation. The right panel shows the mean naming latencies for the high and low agreement pictures, with error bars representing +1 standard deviation from the mean.

On average, participants responded 885 ms after picture onset (see Figure 3) and were faster to name pictures with HA (*M* = 827 ms, *SD* = 125 ms) than those with LA (*M* = 954 ms, *SD* = 145 ms; *b* = −130.42, *SE* = 14.99, *t* = −8.70), replicating Experiment 1. We also found a significant effect of presentation—participants’ naming latencies decreased with each presentation (Presentation 1: *M* = 958 ms, *SD* = 121 ms; Presentation 2: *M* = 866 ms, *SD* = 134 ms; Presentation 3: *M* = 940 ms, *SD* = 840 ms; *b* = −60.17, *SE* = 6.70, *t* = −8.98, for the main effect of presentation). This finding replicates previous studies showing repetition priming during picture naming (e.g., Griffin & Bock, 1998), and suggests that participants found it easier to access a picture’s name with each presentation.

In addition, we found an interaction between NA and presentation (*b* = 43.04, *SE* = 6.80, *t* = 6.33). We followed up this interaction using the emmeans package (version 1.8.7) to compute simple pairwise comparisons at each presentation level with the Bonferroni corrections. The model returned positive estimates because it subtracted the LA effect from the HA effect on each presentation. Participants were faster to name pictures with HA than LA on all three presentations (Presentation 1: *b* = 172.90, *SE* = 18.86, *p* < .001; Presentation 2: *b* = 130.40, *SE* = 18.90, *p* < .001; Presentation 3: *b* = 87.80, *SE* = 21.0, *p* *<* .001), but the difference in latencies for the HA and LA pictures decreased with each presentation because LA pictures benefitted from repetition more than HA pictures.

#### Comparing the NA effect with and without familiarisation

Finally, we compared the NA effect in Experiment 2 (with familiarisation) to Experiment 1 (without familiarisation). We first determined whether participants were more likely to produce modal names with familiarisation (Experiment 1) than without familiarisation (Experiment 2) by fitting a generalised linear mixed-effects model. For comparability across experiments, we included only the first presentation of the pictures in Experiment 2 and we defined the modal name using the BOSS norms, given that participants in Experiment 2 were familiarised with this name. Name type was coded binomially (1 = modal; 0 alternative) and was predicted by NA (reference level: low vs. high), experiment (reference level: no familiarisation vs. familiarisation), and their interaction. Both predictors were contrast-coded (−0.5, 0.5). We fitted the model using a binomial family with the maximal random effects structure, including by-participant random effects for NA and by-item random effects for the experiment. As in our previous analyses, correlations among random effects were set to zero to aid convergence.

Participants were more likely to produce the modal name for HA pictures (*M* = 98.32%, *SD* = 2.07%) than LA pictures (*M* = 70.57%, *SD* = 25.49%; *b* = 3.96, *SE* = 0.25, *p* < .001), and they were more likely to produce the modal names in Experiment 2 (with familiarisation; *M* = 96.88%, *SD* = 3.22%) than in Experiment 1 (without familiarisation; *M* = 71.00%, *SD* = 5.26%; *b* = 2.99, *SE* = 0.25, *p* < .001). There was also an interaction between NA and experiment (*b* = −1.01, *SE* = 0.47, *p* = .03). We followed up this interaction using the emmeans package to compute simple pairwise comparisons for an effect of experiment at each level of NA with the Bonferroni corrections. This analysis showed that there was a significant effect of familiarisation for both HA and LA pictures, but this effect was larger for the LA pictures (*b* = −3.50, *SE* = 0.22, *p* < .001) than the HA pictures (*b* = −2.49, *SE* = 0.43, *p* < .001). Thus, participants were more likely to produce modal names after familiarisation, particularly when naming LA pictures.

To test whether the NA effect was reduced by familiarisation, we analysed naming latencies using a linear mixed-effects model with the same model structure as the generalised analysis. For comparability across the experiments, we included only modal name responses and again focused on the first presentation of the pictures in Experiment 2. As in our previous analyses, participants were faster when naming HA (*M* = 930 ms, *SD* = 152 ms) than LA pictures (*M* = 1,144 ms, *SD* = 214 ms; *b* = −209.22, *SE* = 22.56, *t* = −9.27). They were also faster when naming pictures on their first presentation in Experiment 2 after familiarisation (*M* = 958 ms, *SD* = 121 ms) than in Experiment 1 without familiarisation (*M* = 1,079 ms, *SD* = 186 ms; *b* = −172.54, *SE* = 33.00, *t* = −5.23). There was also an interaction between NA and experiment (*b* = 98.44, *SE* = 31.94, *t* = 3.08) showing that the NA effect was stronger in Experiment 1 than in the first presentation of Experiment 2, consistent with the individual experiment analyses.

In sum, these data show that familiarisation led to a more homogeneous set of responses for the LA items. Familiarisation also reduced the NA effect on naming latencies, but it did not remove it completely. Thus, the difficulty of selecting between alternative names remained.

## General discussion

In two picture-naming experiments, we investigated (1) whether speakers consider a range of names when naming pictures with LA compared to those with HA; and (2) whether previous experience through stimulus familiarisation and repeated naming affected the NA effect by altering the extent to which speakers considered each of these names. In Experiment 1, participants were faster to name HA pictures than LA pictures when they were not familiarised with the pictures or their names, replicating earlier studies (e.g., Barry et al., 1997). Importantly, participants were faster to produce modal than alternative names for LA pictures. As we discussed in the Introduction, earlier studies on NA have not compared naming latencies for these two name types. The advantage for modal names in our study is consistent with the idea that speakers consider a range of possible names for LA pictures and are biased towards selecting the modal name, which is preferred by the wider population (e.g., Balatsou et al., 2022). In Experiment 2, participants were again faster to name HA than LA pictures, even when they were familiarised with the pictures. Although there was still an NA effect when participants named the pictures for the first time, the effect was reduced in comparison to Experiment 1 and was further reduced with each repetition of the picture. Thus, familiarisation and repetition reduced the NA effect but did not eliminate it, suggesting that current word activation levels were affected by previous activity in the lexical network (e.g., Oppenheim et al., 2010).

Participants were slower to produce alternative than modal names for LA pictures, even when they were not familiarised with the pictures and could thus select the name that they found most appropriate or could retrieve most easily. One account of this difference is that alternative names were intrinsically less accessible than modal names because they took more time to select and plan. For example, the modal name *bike* might be faster to access than the alternative name *racing bike* because it is shorter and more frequent. We found that the advantage of modal over alternative names persisted even when we controlled for the word frequency of the names, which argues against this proposal. However, there may be other properties of the modal names that made them faster to access than alternative names and we cannot rule out this possibility.

An alternative view, consistent with Balatsou et al. (2022), is that the advantage for modal names arose because speakers considered a range of possible names for LA pictures and were biased towards selecting the modal name, which is preferred by the wider population. To elaborate, one can think of lexical selection as governed by multiple constraints, including the speakers’ own preference for a name and their knowledge of the community’s preferences. When speakers name a picture with HA, there is only a single strong candidate name and so naming latencies are fast. When speakers name a picture with LA, they have to decide between multiple candidate names, which requires some processing time and leads to longer naming latencies than for HA pictures. When the speaker’s preference matches the population-level preference, the modal name is selected and the response is relatively fast (but not as fast as for pictures with HA). But, when the speaker’s preference mismatches the population-level preference, additional time is required to resolve the conflict between them. Consequently, the speaker will either produce the modal name with a relatively long latency or the alternative name also with a long latency. As a result, naming latencies will be on average longer for alternative than modal names.

We also found that the NA effect was reduced by familiarisation and item repetition in Experiment 2. In our familiarisation protocol, participants were first exposed to the pictures and their names and then had to type a name in response to each picture. Thus, familiarisation could facilitate several components of the picture recognition and name retrieval process. In our working model, picture naming involves visual and conceptual processes leading to the identification of the depicted object and the selection of a lexical concept and a lemma (the grammatical representation of the object name), the retrieval of the morpho-phonological form of the object name, the retrieval of the articulatory commands, and finally the articulation of the name (e.g., Indefrey, 2011; Indefrey & Levelt, 2004). There is a large literature on repetition priming in picture naming (see, e.g., Francis, 2014, for a review), which points to early processes (picture recognition and lemma selection) as the primary origin of repetition priming effects in picture naming (e.g., Tsuboi et al., 2021). However, participants in our study typed the object names in the familiarisation phase, which likely involved the retrieval of their orthographic and phonological forms (e.g., Bonin & Fayol, 2000). Thus, phonological encoding may have also been facilitated.

Regardless of which processes were facilitated by familiarisation, LA pictures benefitted from familiarisation and repetition more than HA pictures. This finding is consistent with theories of lexical access that propose that the accessibility of a word on a particular naming episode is affected by whether it was activated and selected on a previous episode (e.g., Oppenheim et al., 2010). When participants named an LA picture for the first time, they activated multiple plausible names (e.g., *bike* and *racing bike*). They then selected a particular name for production (e.g., *bike*), which strengthened the connection between the conceptual input and the lexical node for that name (*bike*) while simultaneously weakening the connection to the alternate (*racing bike*). As a result, the alternate (*racing bike*) was relatively less strongly activated when participants named the picture a second time, thus making it easier for the participants to produce the target (*bike*) and reducing the NA effect. This finding adds to the body of literature assessing changes in the lexical network through recent activation and selection of items (e.g., Kurtz et al., 2018; Wöhner et al., 2024) and suggests that the activation patterns within the lexicon can be changed by recent experience.

However, the NA effect was not eliminated by familiarisation, which suggests that alternative names for LA pictures are still activated and compete with the modal name even when participants are explicitly asked to use a specific picture name. Participants in our study were familiarised with the modal name. When NA is moderate to low, familiarisation may mean that participants are sometimes asked to produce names they would not necessarily use spontaneously, which may delay responses. An interesting issue for further research is to determine how speakers engage executive control processes to overrule their preference and select the required (familiarised) name. This issue is related to the broader question of how working memory and monitoring processes are engaged when speakers select words appropriate in the current context (e.g., Nozari et al., 2016).

We also found that the NA effect was not eliminated by repetition. Experiment 2 included only three presentations of the materials, so it is possible that the NA effect is eliminated after further repetition. Alternatively, the NA effect may remain regardless of the number of repetitions of the materials. This explanation would again fit with Balatsou et al. (2022), who noted that speakers represent and maintain plausible alternatives in their mental lexicon and use them when required. In line with this argument, it is possible that the alternative name should still be readily available to speakers in other contexts and continue to compete with the modal name even after participants have produced this modal name several times in response to a specific picture. Future research could investigate this issue, providing further insight into the stability of the NA effect.

Our findings have clear methodological implications. First, we found a strong relationship between NA (specifically *H*-values, which represent the spread of names and how often each name is used) in the BOSS norms, which used written name production, and NA in our own data. But although the BOSS norms were good predictors of modal names for HA pictures, they were poor predictors of modal names for LA pictures—only 60% of the LA pictures had the same modal name in the BOSS norms and in our data. Thus, norms are useful for selecting sets of pictures that differ in NA, but they do not predict which names speakers will spontaneously use for LA pictures; at best, they predict which set of two or three names is likely to be used.

Second, familiarisation and item repetition reduced the NA effect. Thus, if variation in NA is a nuisance variable whose impact on naming latencies should be minimised, then participants should be familiarised with the pictures and their names, which will create a uniform set of responses and minimise any NA effects. In contrast, if NA is a dependent variable of interest whose effect should be maximised, then participants should not be familiarised with the materials and the items should not be repeated. When participants are not familiarised with the materials, they will often produce alternatives to the modal names, especially for LA pictures. To minimise data loss, we recommend recording the latencies of all responses and either pooling all responses or including the type of response (modal vs. alternative) in the analysis.

## Conclusion

To conclude, we investigated whether speakers considered a range of names when naming pictures with LA compared to those with HA, and whether previous experience through stimulus familiarisation and repeated naming affected the NA effect by altering the extent to which speakers considered each of these names. Participants were faster to name HA pictures, which have one plausible name (e.g., *arm*) than LA pictures, which have multiple plausible names (e.g., *bike* and *racing bike*). Importantly, participants were faster to produce modal than alternative names for LA pictures, consistent with the commonly held, but rarely tested, idea that speakers consider a range of possible names for LA pictures. Both familiarisation and repetition reduced the NA effect but did not eliminate it. These results point to a certain malleability of the lexical network, but they also show that recent exposure to words does not necessarily eliminate the effects of long-term experience with the words.

## Supplemental Material

sj-docx-1-qjp-10.1177_17470218241274661 – Supplemental material for The influence of familiarisation and item repetition on the name agreement effect in picture naming Supplemental material, sj-docx-1-qjp-10.1177_17470218241274661 for The influence of familiarisation and item repetition on the name agreement effect in picture naming by Ruth E Corps and Antje S Meyer in Quarterly Journal of Experimental Psychology

## Notes

1. We no longer required a beep to mark trial onset as a Frinex update allowed us to begin audio recording as soon as the picture was presented on-screen, enabling us to accurately time-lock the beginning of audio recording to picture onset.
