# Supplementary material for: The influence of familiarisation and item repetition on the name agreement effect in picture naming
Source: Q J Exp Psychol (Hove). 2024 Sep 12;78(7):1487–99. doi: 10.1177/17470218241274661 (PMC12181635; doi:10.1177/17470218241274661)
Supplement: sj-docx-1-qjp-10.1177_17470218241274661 – Supplemental material for The influence of familiarisation and item repetition on the name agreement effect in picture naming [file sj-docx-1-qjp-10.1177_17470218241274661.docx]

Supplementary Material for:

**The influence of familiarisation and item repetition on the name agreement effect in picture naming**

Ruth E. Corps^1,2^ & Antje S. Meyer^2,3^

^1^ Department of Psychology, University of Sheffield

^2^ Psychology of Language Department, Max Planck Institute for Psycholinguistics

^3^ Radboud University

Please address correspondence to:

Ruth Elizabeth Corps

Department of Psychology

219 Portobello, Broomhall

University of Sheffield

Sheffield, S1 4DP

United Kingdom

[r.corps@sheffield.ac.uk](mailto:r.corps@sheffield.ac.uk)

Table A1: Full list of high and low agreement pictures used in Experiments 1. For each picture, we provide the modal name from the BOSS database and the modal name from Experiment 1. These names are in Dutch, but we also provide the English filename of the picture in the database. Note that Experiment 2 used the same items, but they were categorised as high or low agreement using the original BOSS norms.

| Name Agreement | BOSS Modal Name | Experiment 1 Modal Name | English Picture Name | Category |
| --- | --- | --- | --- | --- |
| Low | Aanhangwagen | Aanhangwagen | Boxtrailer | Vehicle |
|  | Aap | Aap | Gorilla | Animal |
|  | Arend | Arend | Eagle | Animal |
|  | Bankje | Bankje | Parkbench02 | Furniture |
|  | Basket | Basketbalnet | Basketballhoop01 | Sports |
| (originally high) | Blad | Blad | Leaf02a | Plant |
|  | Blauwe-kaas | Kaas | Bluecheese | Food |
|  | Bloem | Bloem | Daffodil | Plant |
|  | Boterham | Boterham | Breadslice | Food |
| (originally high) | Brood | Brood | Bread | Food |
|  | Buis | Buis | Pipe | Other |
|  | Cabrio | Cabrio | Convertible | Vehicle |
|  | Camera | Camera | Camera01a | Electronics |
|  | Deur | Deur | Doubledoors | Part of building |
|  | Draad | Garen | Thread02 | Sewing box |
|  | Drumstokken | Drumsticks | Drumstick | Musical instrument |
|  | Dvd-hoes | Cd-hoesje | Dvdcase01 | Electronics |
|  | Eisnijder | Eiersnijder | Eggslicer | Kitchen utensils |
|  | Feesthoedje | Feestmuts | Partyhat | Party decoration |
|  | Fietsslot | Slot | Bikelock | Part of vehicle |
|  | Flesje | Flesje | Waterbottle01b | Kitchen utensils |
|  | Flesopener | Flesopener | Bottleopener01 | Kitchen utensils |
| (originally high) | Gereedschapskist | Gereedschapskist | Toolbox02 | Tools |
|  | Haarclip | Haarklem | Hairclip03 | Accessory |
|  | Hakmes | Mes | Cleaver02 | Outdoor |
|  | Honkbalknuppel | Honkbalknuppel | Baseballbat | Sports |
| (originally high) | Hyena | Hyena | Hyena | Animal |
|  | Ijskrabber | Ijskrabber | Icescraper | Tools |
|  | Jetski | Jetski | Jetski | Vehicle |
|  | Kaarsje | Kaars | Birthdaycandle | Party decoration |
|  | Kaart | Kaart | Aceofdiamond | Outdoor |
|  | Kaas | Kaas | Softcheese | Food |
|  | Kampvuur | Vuur | Campfire | Outdoor |
|  | Kast | Kast | Dresser02 | Furniture |
|  | Kentekenplaat | Nummerbord | Licenseplate | Part of vehicle |
|  | Keukenrol | Keukenrol | Papertowel | Kitchen utensils |
|  | Kinderfiets | Fiets | Kidbicycle | Vehicle |
|  | Klavertje-drie | Klavertje-drie | Clover | Plant |
|  | Kliko | Kliko | Garbagebin | Other |
|  | Kopje | Mok | Mug01 | Kitchen utensils |
|  | Lynx | Kat | Lynx01 | Animal |
|  | Map | Map | Binder03b | Office supplies |
|  | Meetlint | Meetlint | Measuringtape01 | Sewing box |
| (originally high) | Mier | Mier | Ant | Animal |
|  | Mixer | Mixer | Electricmixer | Kitchen utensils |
| (originally high) | Molen | Molen | Windmill | Building |
|  | Munten | Munten | Coin01b | Valuables |
|  | Nintendo | Nintendo | NintendoDS | Electronics |
|  | Overhemd | Overhemd | Shirt02 | Clothing |
|  | Pan | Pan | Fryingpan02a | Kitchen utensils |
|  | Pantoffel | Slof | Slipper01b | Clothing |
|  | Papieren-vliegtuig | Papieren-vliegtuigje | Paperairplane | Toys |
|  | Poort | Hek | Entrancegate | Part of building |
|  | Prittstift | Prittstift | Gluestick | Office supplies |
|  | Put | Put | Manholecover | Other |
|  | Rookmelder | Rookmelder | Smokedetector02 | Part of building |
|  | Schaakbord | Schaakbord | Chessboard | Toys |
|  | Schaal | Kom | Bowl02a | Kitchen utensils |
|  | Schakelaar | Schakelaar | Lightswitch01 | Other |
|  | Scheermesje | Scheermesje | Razor01 | Other |
|  | Shirt | Polo | Poloshirt | Clothing |
|  | Spaarpot | Spaarpot | Piggybank | Valuables |
|  | Speelgoedpaard | Paard | Toyanimal05 | Toys |
|  | Spin | Spin | Tarantula | Animal |
|  | Tablet | Tablet | Ipad02 | Electronics |
|  | Tafeltennistafel | Tafeltennistafel | Pingpongtable | Sports |
|  | Tasje | Tasje | Giftbag01a | Other |
|  | Tosti-ijzer | Tosti-ijzer | Paninigrill | Kitchen Utensils |
|  | Trapleuning | Hek | Handrail | Part of building |
|  | Tuinhuisje | Tuinhuisje | Shed02 | Building |
|  | Videocamera | Videocamera | Videocamera01a | Electronics |
|  | Vikinghelm | Vikinghelm | Vikingmask01b | Other |
|  | Vogelhuisje | Vogelhuisje | Birdhouse | Outdoor |
|  | Waterlelie | Bloem | Waterlily | Plant |
| (originally high) | Worst | Worst | Sausage | Food |
|  | Zoutvaatje | Zout | Saltshaker03a | Kitchen utensils |
|  | Zwembroek | Broek | Swimsuit | Clothing |
| High | Aardappel | Aardappel | Potato02b | Food |
|  | Aardbei | Aardbei | Strawberry | Food |
|  | Anker | Anker | Anchor | Part of vehicle |
|  | Avocado | Avocado | Avocado01 | Food |
|  | Ballon | Ballon | Balloon01b | Party decoration |
|  | Batterij | Batterij | Battery02b | Electronics |
|  | Bel | Bel | Callbell | Other |
|  | Brood | Brood | Bread | Food |
|  | Cactus | Cactus | Cactus | Plant |
|  | Champignon | Champignon | Mushroom01 | Food |
|  | Croissant | Croissant | Croissant01 | Food |
|  | Donut | Donut | Donut | Food |
| (originally low) | Douchecabine | Douche | Shower | Part of building |
|  | Eend | Eend | Duck01 | Animal |
|  | Elleboog | Elleboog | Elbow | Body part |
|  | Envelop | Envelop | Envelope03a | Office supplies |
|  | Fontein | Fontein | Parkfountain | Other |
|  | Frisbee | Frisbee | Frisbee | Toys |
|  | Giraffe | Giraffe | Giraffe | Animal |
| (originally low) | Gitaar | Gitaar | Bassguitar | Musical instrument |
|  | Gordijn | Gordijn | Curtain | Home decoration |
|  | Gum | Gum | Eraser | Office supplies |
|  | Hand | Hand | Hand01b | Body part |
|  | Horloge | Horloge | Watch02a | Accessory |
|  | Hyena | Hyena | Hyena | Animal |
|  | Kalender | Kalender | Calendar | Other |
|  | Kapstok | Kaptstok | Coatrack | Home decoration |
|  | Kasteel | Kasteel | Castle | Building |
|  | Kluis | Kluis | Safe | Valuables |
|  | Knie | Knie | Knee | Body part |
|  | Knikker | Knikker | Marble | Toys |
|  | Knoop | Knoop | Button01 | Sewing box |
|  | Kokosnoot | Kokosnoot | Coconut | Food |
|  | Kompas | Kompas | Magneticcompass | Outdoor |
|  | Kraan | Kraan | Faucet | Part of building |
|  | Krokodil | Krokodil | Crocodile | Animal |
|  | Kroon | Kroon | Crown | Accessory |
|  | Kussen | Kussen | Pillow01a | Home decoration |
|  | Kwal | Kwal | Jellyfish | Animal |
|  | Laars | Laars | Boot02b | Clothing |
|  | Laptop | Laptop | Laptop01a | Electronics |
|  | Lieveheersbeestje | Lieveheersbeestje | Ladybug03 | Animal |
|  | Lolly | Lolly | Lollipop04 | Food |
|  | Medaille | Medaille | Medal02b | Sports |
|  | Neus | Neus | Nose | Body part |
|  | Nietjes | Nietjes | Staple | Office supplies |
| (originally low) | Pad | Kikker | Toad | Animal |
|  | Paraplu | Paraplu | Umbrella04 | Outdoor |
|  | Passer | Passer | Mathcompass | Office supplies |
| (originally low) | Pasta | Pasta | Fusilli03a | Food |
|  | Pleister | Pleister | Bandaid01 | Other |
|  | Pompoen | Pompoen | Pumpkin | Food |
|  | Printer | Printer | Printer02 | Electronics |
|  | Rietje | Rietje | Straw | Kitchen utensils |
|  | Ring | Ring | Ring01 | Accessory |
|  | Schaar | Schaar | Scissors01 | Office supplies |
|  | Schelp | Schelp | Seashell01 | Other |
|  | Schilderij | Schilderij | Painting | Home decoration |
|  | Schoorsteen | Schoorsteen | Chimney | Part of building |
|  | Schroef | Schroef | Screw02 | Tools |
|  | Schroevendraaier | Schroevendraaier | Screwdriver04b | Tools |
|  | Sigaret | Sigaret | Cigarette | Other |
|  | Spaghetti | Spaghetti | Spaghetti01 | Food |
|  | Spiegel | Spiegel | Mirror02 | Home decoration |
| (originally low) | Stoel | Stoel | Outdoorchair | Furniture |
|  | Tent | Tent | Tent | Outdoor |
|  | Touw | Touw | Rope03 | Other |
|  | Triangel | Triangel | Triangle | Musical instrument |
|  | Trompet | Trompet | Trumpet | Musical instrument |
|  | Varken | Varken | Pig | Animal |
|  | Vleermuis | Vleermuis | Bat | Animal |
|  | Vlieger | Vlieger | Kite | Toys |
|  | Vlinder | Vlinder | Butterfly | Animal |
|  | Wolk | Wolk | Cloud | Other |
|  | Zeehond | Zeehond | Seal | Animal |

Table A2: List of categories and the number of items in each category for high and low agreement pictures used in both experiments.

| Category | High agreement | Low agreement |
| --- | --- | --- |
| Accessory | 3 | 1 |
| Animal | 11 | 5 |
| Body part | 4 | 0 |
| Building | 2 | 1 |
| Clothing | 1 | 4 |
| Electronics | 3 | 5 |
| Food | 12 | 4 |
| Furniture | 0 | 3 |
| Home decoration | 5 | 0 |
| Kitchen utensils | 1 | 10 |
| Musical instrument | 2 | 2 |
| Office supplies | 5 | 2 |
| Other | 8 | 7 |
| Outdoor | 3 | 4 |
| Part of building | 2 | 5 |
| Part of vehicle | 1 | 2 |
| Party decoration | 1 | 2 |
| Plant | 2 | 3 |
| Sewing box | 1 | 2 |
| Sports | 1 | 3 |
| Tools | 3 | 1 |
| Toys | 3 | 3 |
| Valuables | 1 | 2 |
| Vehicle | 0 | 4 |
